# Central dopamine receptors: Radiotracers unveiling the Role of dopaminergic tone in obesity

**DOI:** 10.1007/s00109-024-02501-0

**Published:** 2024-12-04

**Authors:** Marta Lapo Pais, Joana Crisóstomo, Antero Abrunhosa, Miguel Castelo-Branco

**Affiliations:** 1 Coimbra Institute for Biomedical Imaging and Translational Research (CIBIT), University of Coimbra, Coimbra, Portugal; 2 Institute for Nuclear Sciences Applied to Health (ICNAS), University of Coimbra, Coimbra, Portugal; 3 Faculty of Science and Technology, University of Coimbra, Coimbra, Portugal; 4 Champalimaud Research, Champalimaud Foundation, Lisbon, Portugal; 5 Faculty of Medicine, University of Coimbra, Coimbra, Portugal

**Keywords:** Central dopamine receptors, Obesity, Radiotracers, Dopaminergic tone, Molecular neuroimaging

## Abstract

Brain dopamine type 2 and 3 receptors (D2/3R) have been postulated to play a role in obesity. However, results from molecular neuroimaging studies exploring these receptors in obesity are not consensual. These inconsistencies may be due to the distinct characteristics of radiotracers that confound the interpretation of D2/3R assessment. Only three meta-analyses reported their results across radiotracers. Although all agree that obesity severity influences D2/3R availability, results vary for [^11^C]raclopride. Further, D2/3R assessment has been commonly interpreted as reflecting receptor density or availability. An alternative interpretation could be related to changes in endogenous central dopaminergic tone. The main question is whether the hypothesis of a quadratic relationship between dopaminergic tone and degree of obesity is suitable for the distinct characteristics of radiotracers. To answer this question and clarify the role of dopaminergic tone in obesity, we systematically reviewed this issue across radiotracers. Out of 514 articles, 15 articles were selected for review. Besides obesity severity, this study highlights the influence of radiotracer characteristics when assessing D2/3R. The tested hypothesis proved to be more suitable for radiotracers more susceptible to endogenous dopamine or with a lower affinity to D2/3R, supporting the quadratic relationship between dopaminergic tone and degree of obesity. While the role of D2/3R density in obesity may be relevant, dopaminergic tone seems to have a greater impact on the obesity-related differences found in these receptors. Finally, neuropsychological factors should be tested in addition to body mass index, as they may better reflect altered brain dopaminergic function.

**Graphical Abstract:**

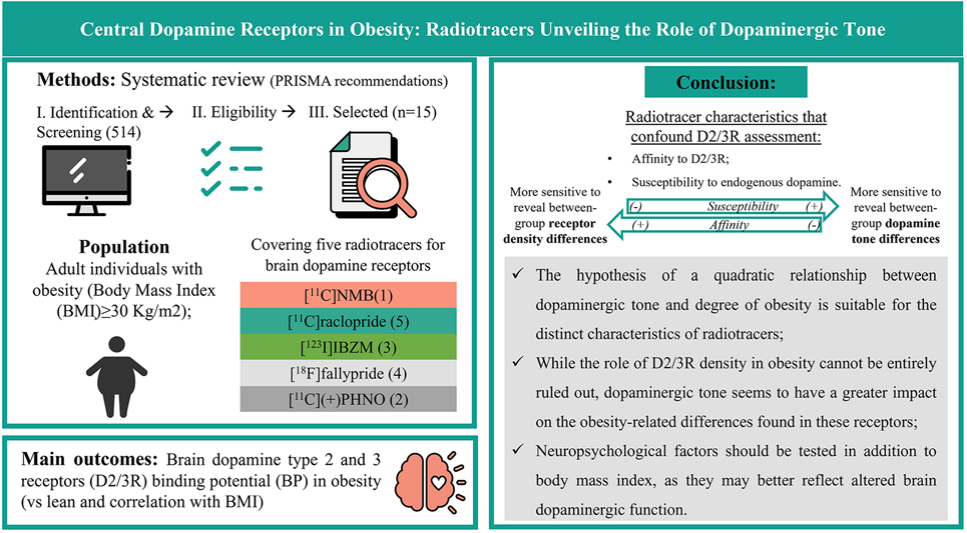

## Introduction

Obesity is a multi-causal disease resulting from an imbalance between energy intake and expenditure [1–3]. As the prevalence of obesity continues to rise, there is a need to search for explanations for why some individuals continue to consume food under conditions of positive energy balance [3, 4].

Two main complex brain circuits coordinate the eating behaviour that impacts body weight. The hedonic circuitry dictates the desire to eat regarding palatability and pleasure for certain foods, which is involved in motivational aspects of energy consumption (reward) [5, 6]. On the other hand, the homeostatic circuitry matches energy intake to energy expenditure to maintain body weight and metabolic function (based on energy needs) [5, 6]. In addition, several neurotransmitters play a role in these brain circuits, such as dopamine, GABA, norepinephrine and serotonin, which are also influenced by several hormones to regulate food intake [7].

Dopamine has received attention in this field, as diminished signalling of this neurotransmitter has been not only associated with obesity but also postulated to contribute to the maintenance of obesity status [8]. Furthermore, food is naturally rewarding and typically acts on the reward pathways in the brain, where dopamine represents the most important neurotransmitter [9]. There are four main domains to target dopamine signalling: (1) dopamine receptors, (2) dopamine release, (3) dopamine synthesis, and (4) dopamine transporters [10]. Dopamine type 2 receptors (D2R) have particular interest in this regard as drugs that block D2R increase appetite and result in significant weight gain [11–13]. Furthermore, imaging studies and genetic analysis have also demonstrated that people with obesity tend to show altered expression of D2R in specific brain areas [14]. Moreover, humans with decreased brain D2R are more vulnerable to compulsive behaviours, including disorders related to food intake [11]. Nuclear medicine imaging techniques such as positron emission tomography (PET) and single-photon emission computed tomography (SPECT) are the most direct ways to visualise and quantify in vivo these receptors in human subjects [3, 10]. Regarding quantification, the binding potential (BP) is the most common outcome measure used as a surrogate measure of receptor density [15]. However, in vivo*,* not all receptors are available for the radiotracer to bind because of occupancy by endogenous ligands. Hence, BP is defined as an integrated metric of receptor availability and radioligand affinity rather than a direct measure of receptor density [3, 16]. Ultimately, it reflects the equilibrium concentration of specific binding as a ratio to some reference: free plasma concentration, total plasma concentration, or nondisplaceable uptake [16]. Even using this same outcome measure, the results of PET and SPECT studies investigating the role of brain dopamine receptors in obesity are not consensual. We propose that these inconsistent results may be due to the distinct characteristics of each radiotracer, including its affinity for dopamine receptors and interactions with endogenous dopamine. Radiotracers with a higher affinity for a given receptor will yield a higher BP than the ones with a lower affinity [3]. On the other hand, when a radiotracer competes with endogenous dopamine for receptor binding, higher endogenous concentrations of this neurotransmitter may decrease BP values [3]. To our knowledge, only three meta-analyses on brain dopamine receptors in obesity investigate their results across radiotracers [17–19]. Ribeiro et al*.* (2023) [18] report no striatal D2R availability differences between obesity and controls for the majority of the radiotracers, namely, [^11^C]raclopride (2 studies included [7, 20]), [^18^F]fallypride (2 studies included [21, 22]) and N-[^11^C]methyl)benperidol ([^11^C]NMB) (2 studies included [23, 24]), but a significant difference was found for [^123^I]iodobenzamide ([^123^I]IBZM) (3 studies included [25–27]). Another meta-analysis, restricted to [^11^C]raclopride (5 studies included [7, 17, 28–30]), also failed to establish a strong association between striatal D2R availability and body mass index (BMI) [17]. Finally, Pak et al*.* (2023) [19] reported that in subjects with a BMI of 25 kg/m^2^ or higher, D2R availability was negatively associated with BMI for [^11^C]raclopride (4 studies included [7, 17, 29, 31]) but was positively associated with BMI for [^11^C]( +)PHNO (2 studies included [31, 32]). These meta-analyses have points of conclusion in common, namely that the degree of obesity severity influences Dopamine type 2 and 3 receptors (D2/3R) assessment [17–19]. However, results vary for [^11^C]raclopride, and these meta-analyses are limited to the number of studies included across radiotracers and variations in sample characteristics [17–19]. Further, data on receptor availability have been commonly interpreted as reflecting D2/D3 availability or density despite the possibility of an alternative interpretation, namely alterations in the dopaminergic tone [33]. As a physiological concept, dopaminergic tone refers to the endogenous dopamine concentration in the brain and results from the balance between dopamine release and uptake [34]. In a review, Horstmann et al. (2015) proposed a quadratic relationship between dopaminergic tone and the degree of obesity [33]. Nevertheless, although the author alerted that the D2/3R assessment ascribed to receptor density or dopaminergic tone depends on the radiotracer used, this hypothesis was not tested across radiotracers [33]. The main question posed by this review is whether the quadratic relationship between dopaminergic tone and degree of obesity proposed by Horstmann et al. (2015) is suitable for the distinct characteristics of radiotracers [33]. To answer this question, we propose to perform a systematic review of the literature on this topic across radiotracers. It is important to note that while microdialysis is a direct but invasive measure of dopamine tone, PET is an indirect measure [34]. Even so, this approach will allow insights into a clearer distinction between receptor density and dopaminergic tone roles in obesity. Throughout this work, to facilitate the interpretation of the results, obesity will be split into three classes regarding severity: class I obesity (30–34.9 kg/m^2^), class II obesity (35–39.9 kg/m^2^) and class III or higher: $\ge$ 40 kg/m^2^.

## Methods

### Identification and search strategy

**Fig. 1:**
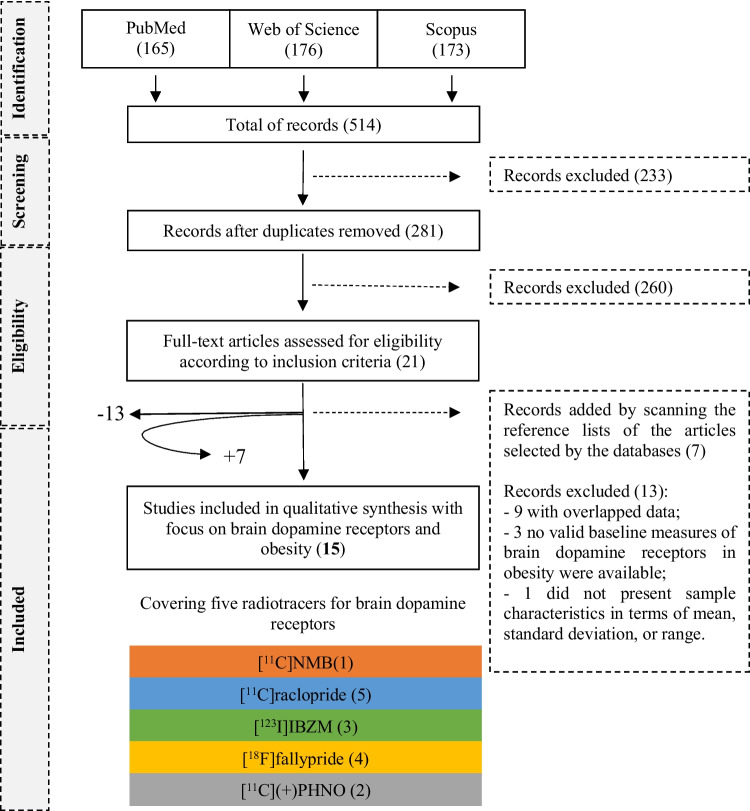
Flow chart of the literature search. We performed a systematic review according to PRISMA guidelines, and from an initial pool of 514 articles, 21 met the inclusion criteria, 13 were excluded and 7 were added by scanning the reference lists of the articles selected by the databases. Colored boxes contain the number of retained articles per radiotracer. [^11^C]NMB = (N-[^11^C]methyl)benperidol; [^123^I]IBZM = [^123^I]iodobenzamide

The methodology presented here was registered in the Prospero Platform with an ID number of CRD42023470887. Recommendations for the systematic review were followed as closely as possible to the PRISMA statement for reporting systematic reviews and meta-analyses of studies that evaluate healthcare intervention [35]. A scheme of the structured qualitative review is presented in Fig. 1. Studies were identified by three searching electronic databases (PubMed, Web of Science and Scopus) and by scanning the reference lists of the articles selected by these databases. The last search was run on October 1st, 2024. During the search strategy, the following filters were imposed: written in English, open access, human species (just for PubMed) and publication date from January 1st, 2000, to October 1st, 2024. Considering the limited number of articles available on this topic, we conducted the search terms as broadly as possible to ensure that no potentially relevant articles were excluded a priori. As such, search terms included "obesity", "dopamine", and "dopamine receptors". The search strategy and the number of articles identified are presented in Table 1. Other papers were used to contextualise and discuss the selected studies.

**Table 1:** Databases used, search strategy and the number of articles identified

Databases	Search	Number of articles
PubMed	(obesity[MeSH Major Topic]) AND ((dopamine) OR ("dopamine receptors"));	165
Web of Science	(TI = (obesity)) AND AB = (dopamine OR "dopamine receptors");	176
Scopus	TITLE (obesity) AND ABS (dopamine OR "dopamine receptors");	173

MeSH = Medical Subject Headings (controlled vocabulary used for indexing articles for PubMed); TI = title and AB = abstract (controlled vocabulary used for indexing articles for Web of Science); ABS = abstract (controlled vocabulary used for indexing articles for Scopus)

### Selection of studies

The inclusion criteria included PET or SPECT clinical studies that examined brain dopamine receptors in obesity. After assessing 21 full-text articles for eligibility according to inclusion criteria, seven papers were added by scanning the reference lists of the articles selected by the databases [22, 25–27, 29, 36, 37] and 13 were excluded [17, 24, 38–48]. Nine of them were excluded because they included overlap data [17, 24, 40, 42–47]. In these cases, we selected the most relevant publication among the pool of articles with overlapping data. We excluded three other articles because no valid baseline measures of brain dopamine receptors in obesity were available [39, 41, 48]. Finally, the last one was excluded because it did not present sample characteristics in terms of mean, standard deviation, or range [38] (see Fig. 1 for details).

A total of 514 articles were screened, and 15 articles were retained. The full texts and references of the remaining 15 articles were assessed in detail. The following information was extracted: radiotracer and sample characteristics, summary of results, authors, and year of publication (see Table 2 for details).

**Table 2:** Studies retained: Radiotracer, sample characteristics, summary of results and year of publication and references

	Sample characteristics						Summary of results		Authors, year/ Ref
	Group	N (F/M)	Age (years) Mean (SD), range	BMI (kg/m^2^) Mean (SD), range		BP in obese (vs lean)		Correlation (BP vs BMI)	
[^18^F]fallypride	Lean	23(11/12)	28, 25.1–30.4	22.4, 21.3–23.5		= in PUT, CAU, NAc		None in PUT, CAU, NAc	Guo et al. 2022/[49]
	Obese	20(10/10)	35, 31.9–38.8	36.1, 34–38.3					
	Lean + obese	33(15/18)	25.8, 18–35	24.8, 19–35				(-) in left CAU and right AMG and none in PUT, vSTR, SN, right CAU and left AMG	Kessler et al*.* 2014/[36]
	Lean + obese	130(72/58)	35.6(18.2)	25.5(4.8), < 18.5- > 40				( +) in PUT and none in vSTR, MB and CAU; age $>$ 30: ( +) in MB, PUT and vSTR; age $<$ 30: none for all regions	Dang et al*.* 2016/[22]
	Lean	8(8/0)	41(9)	22(3)		↑ PUT, vSTR and CAU		( +) in CAU and PUT	Dunn et al*.* 2019/[8]
	Obese	18(18/0)	39(8)	39(6)					
[^11^C]raclopride	Lean	12(6/6)	33.2(8)	25(3)		↓ STR			Volkow et al*.* 2008/[50]
	Obese	10(5/5)	35.9(10)	51(5)					
	Lean	5(5/0)	21.8	21.3		= in vSTR, PUT, CAU			Steele et al*.* 2010/[29]
	Obese	5(5/0)	32.2(7.33),20–38	45.2(5.89),40–53					
	Obese w/BED	10(8/2)	38.5(13.3), 21–54	43.4(13.5), 30–65		= between obese w/BED and without BED		None in CAU	G. J. Wang et al*.* 2011/[51]
	Obese	8(5/3)	41.8(8.9), 28–56	36.5(9.4), 31–59					
	Lean	14(14/0)	44.86(12.9)	22.7(2.9)		= in vSTR, dCAU, PUT		None in vSTR, dCAU, PUT	Karlsson et al*.* 2015/[17]
	Obese	13(13/0)	39.08(10.7)	41.9(3.9), 37.1–49.3					
	Lean	5(5/0)	24.0(4.18)	21.8(0.16)		↓ in vSTR			Carnell et al*.* 2023/[52]
	Obese	6(6/0)	43.5(8.02)	41.8(5.48)					
[^11^C]( +)PHNO	Lean + obese	12(2/10)	28.3(6.0), 20 – 37	27.9(4.5), 21.5 – 36.5				( +) in the right dCAU and none in left dCAU	Cosgrove et al*.* 2015/[37]
	Lean	14(4/10)	34.9(10.2)	22.3(1.8)		↑ in the SN/VTA, vSTR, and PAL and = in the AMG, CAU, HYPO, PUT, THL		( +) in the SN/VTA, vSTR, and PAL and none in the AMG, CAU, HYPO, PUT, THL	Gaiser et al*.* 2016/[32]
	OW	14(1/13)	36.7(11.5)	27.2(1.3)					
	Obese	14(4/10)	37.0(10.1)	35.3(4.5)					
[^11^C]NMB	Lean	20(15/5)	28.6(5.3), 21–39.7	22.4(2.4), 18.6–27.7		= in STR		None in STR	Eisenstein, Gredysa, et al*.* 2015/[23]
	Obese	27(23/4)	31.5(6.61), 20–40.9	39.90(4.76), 33.2–51					
[^123^I]IBZM	Lean	15(15/0)	28.8(10.4), 20–60	21.7(2.1), 19.5–27.6		↓ in STR			de Weijer, et al*.* 2011/[25]
	Obese	15(15/0)	37.8(7.0), 26–49	46.8(6.5), 38.7–61.3					
	Lean	15(15/0)	38.5(5.6)	21.8(1.8), 18.5–24.9		↓ in STR		None in STR	van de Giessen et al*.* 2014/[27]
	Obese	15(15/0)	36.3(4)	42.9(4.9), 36.3–56.5					
	Lean	11(11/0)	40.5(4)	21.9(2)		↓ in STR		None in STR	Van der Zwaal et al*.* 2016/[26]
	Obese	11(11/0)	44.3(6)	45.2(6.7), 38.7–61.3					

Sample characteristics include N = sample size (females and males ratio (F/M)), age in years (mean (SD), range) and BMI = body mass index in kg/m^2^ (mean (SD), range) per group. AMG = amygdala, BED = Binge eating disorder; CAU = caudate nucleus, HYPO = hypothalamus, MB = dopaminergic midbrain (substantia nigra (SN) and ventral tegmental area (VTA)), NAc = nucleus accumbens, OW = overweight, PAL = pallidum, PUT = putamen, STR = striatum, THL = thalamus, TL = temporal lobe, which can be specified by prefixes “d” = dorsal, “l” = lateral and “v” = ventral

## Results and Discussion

As expected, distinct findings regarding dopamine type 2 and 3 receptors (D2/3R) BP obesity-related differences are reported across the 15 articles retained (see Table 2).

Four studies using PET-radiotracers ([^18^F]fallypride (n = 2) and [^11^C]( +)PHNO (n = 2)) indicated higher BP of these receptors in obesity [8, 22, 32, 37]. On the contrary, six studies, three using PET-radiotracers ([^18^F]fallypride (n = 1) and [^11^C]raclopride (n = 2)) and three using the SPECT-radiotracer [^123^I]IBZM, indicated lower BP [25–27, 36, 50, 52]. In the remaining five studies using PET-radiotracers ([^11^C]NMB (n = 1), [^11^C]raclopride (n = 3) and [^18^F]fallypride (n = 1)), no obesity-related differences were reported [17, 23, 29, 49, 51]. Overall investigation on this topic has proposed that lower striatal D2/3R BP may be characteristic of higher degrees of obesity (class III or higher: $\ge$ 40 kg/m^2^) [3, 10, 18]. On the other hand, for overweight, class I/II obesity, evidence points towards higher striatal D2/3R BP [3, 10]. Only 9 out of these 15 studies retained showed results supporting this hypothesis [8, 22, 25–27, 32, 37, 50, 52]. We propose that these discrepancies could be partly explained by the radiotracer used and the influence of its characteristics on the outcome measure, the BP. Horstmann et al. (2015) suggested that the relationship between brain dopamine receptors and the degree of obesity was linked to the dopaminergic tone [33]. In an attempt to explain these discrepancies and clarify the role of dopaminergic tone in obesity, we will closely examine the results of these 15 studies retained across radiotracer characteristics. See Table 3 for details about the characteristics of the radiotracers used by the retained studies.

**Table 3:** Characteristics of the radiotracers used by the retained studies

Radiotracer	Susceptibility^a^ to endogenous dopamine	Affinity^a^ to dopamine receptors	
		D2R	D3R
[^11^C]NMB	-	+ +	-
[^18^F]fallypride	+	+ + +	+ + +
[^11^C]raclopride	+	+ +	+ +
[^123^I]IBZM	+	+	+
[^11^C]( +)PHNO	+ +	+ +	+ + ( +)

D2R = dopamine type 2 receptors, D3R = dopamine type 3 receptors, [^11^C]NMB = (N-[^11^C]methyl)benperidol; [^123^I]IBZM = [^123^I]iodobenzamide. ^a^Affinity and susceptibility range from irrelevant ( −) to very high (+ + +). Table adapted from Table 1 of van Galen et al. (2018) [3] and Supplementary Table 2 of Ribeiro et al. (2023) [18]

### Radiotracers with non-displaceable binding

[^11^C]NMB is the only radiotracer of the retained studies that does not compete with endogenous dopamine for binding in D2R. This radiotracer is also the only one shown to have a 200-fold higher affinity for D2R versus dopamine type 3 receptors (D3R) (see Table 3) [23, 53]. Given its characteristics, it is the most suitable radiotracer of the retained studies to understand the role of D2R density in obesity.

#### [^11^C]NMB (n = 1)

Due to overlapping data [24, 45, 46], we were only able to include one [^11^C]NMB PET study in our systematic review [23]. Both the study retained [23] and the meta-analysis of Ribeiro et al*.* (2023) [18] reported no differences in striatal D2R availability between obesity and controls for this radiotracer. These studies have similar sample characteristics, with a mean BMI of the obese group around 40 kg/m^2^ (in the borderline between class II and class III obesity), ranging from class I to class III [23, 24]. Since [^11^C]NMB is not susceptible to endogenous dopamine and presents a high affinity for D2R over D3R, we question whether obesity-related differences are more prominent in D3R. The authors of the study retained [23], as well as those studying overlapping subjects [46], by finding an association between D2R BP_ND_ and characteristics of eating and reward behaviour, concluded that an emotional eating phenotype [46], or monetary reward discounting behaviour[23] might reflect altered D2R function better than the commonly used BMI measure [23, 46]. In the same line of thought, Pepino et al*.* (2016) [45], with overlapping data from these two studies, used sucrose preferences as an alternative to BMI. Although striatal D2R BP_ND_ correlated with sucrose preferences in subjects without obesity, no correlation was found in subjects with obesity [45]. The authors stated that the mechanism underlying this lack of association in subjects with obesity is unknown [45]. These results leave open the hypotheses of a quadratic relationship between dopaminergic tone and the degree of obesity and support the association between the dopaminergic system and motivation eating and reward behaviours rather than BMI alone.

### Radiotracers with displaceable binding

Radiotracers susceptible to endogenous dopamine confound the interpretation of differences in receptor availability with differing levels of dopamine release. Suppose one assumes that obesity is associated with altered striatal dopamine content due to altered dopamine release or uptake. In that case, radiotracers with displaceable binding might lead to altered D2/3R receptor BP in the striatum due to displacement, while [^11^C]NMB would not [24, 32]. Moreover, these radiotracers do not distinguish well between D2R and D3R subtypes as they present a similar high affinity for both dopamine D2R and D3R (see Table 3 for details) [24, 53]. Since D2R and D3R overlap in several brain regions, it became more accepted to classify these radiotracers as D2/3R radioligands [54].

#### [^11^C]raclopride (n = 5)

From the [^11^C]raclopride PET studies retained, three reported that obesity is associated with unaltered D2/3R BP_ND_ in the brain [17, 29, 51], whereas two found lower D2/3R BP_ND_ in obesity [50, 52]. First, we will closely examine those that found no D2/3R BP_ND_ differences for class III obesity when we expect to observe lower striatal D2/3R BP_ND _[17, 29]. Steele et al*.* (2010) [29], using a small sample of 5 females per group, found no D2/3R BP_ND_ significant differences in the ventral striatum (vSTR), putamen and caudate between obese and normal-weight subjects. This study included females with a mean age of 21.8 years old for lean and 32.2 +/- 7.33 years old for obese and a BMI range of obesity between 40-53 kg/m^2^ [29]. Karlsson et al*.* (2015) [17], when investigating a female population with a higher combined mean age of 41.965 $\pm$ 12.412 years old and a BMI range for obesity between 37.1–49.3 kg/m^2^ (class II and III obesity), also found no significant differences in D2/3R BP_ND_ between obese and normal-weight and no correlation with BMI. Although both studies include a mean BMI for obesity in class III, Karlsson et al*.* (2015) [17] include both class II and III obesity, and both studies only investigate females. One of the authors proposed that lowered D2/3R availability in obesity might be an exception restricted to BMI higher than 50 kg/m^2^ rather than a general pathophysiological feature [17]. We add that the lack of differences may also be because Steele et al*.* (2010)[29] only include 5 females per group and Karlsson et al*.* (2015) [17] include obese individuals with a BMI ranging between 37.1 kg/m^2^ and 49.3 kg/m^2^, where class III obesity effects may be cancelling the ones of class II. Moreover, none of the women included in the study of Steele et al*.* (2010) [29] were older than 38 years old. Given the small sample size of women of reproductive age, potential bias from the menstrual cycle cannot be excluded. Finally, G. J. Wang et al. (2011) [51] also reported no D2/3R BP_ND_ differences between obese individuals with and without binge eating disorder. Moreover, although these binding values were not correlated with BMI, they were positively correlated with the binge eating scores in the caudate [51]. This result reinforces the previous hypothesis that factors related to eating behaviours may reflect altered brain D2/3R function better than the commonly used BMI measure. Since this study also included male and female participants from class I to class III obesity [51], we could also argue once again that the effects of class III obesity may potentially overshadow those of class I. On the contrary, the study with the highest mean BMI for the obese group (51.5 kg/m^2^) of all retained studies found lower D2/3R binding values in obesity [50]. The other study that found lower D2/3R binding values in class III obesity included a small number of subjects, 5 healthy-weight and 6 females with obesity [52]. All three meta-analyses investigated [^11^C]raclopride, but distinct results were reported [17–19]. Ribeiro et al. (2023) [18] found no striatal D2/3R availability differences between obesity and controls (2 studies included [7, 20]) [18]. Although G.-J. Wang et al. (2001) [7] included obese individuals with a mean BMI in class III obesity (51.5 kg/m^2^), G.-J. Wang et al. (2014) [20] only included overweight and class I obesity (25–35 kg/m^2^). The lack of findings may result from the heterogeneity in the BMI range of the studies included. Studies using [^11^C]raclopride from different institutions and with more homogenous sample characteristics are missing in this meta-analysis. Karlsson et al. (2015) (5 studies included [7, 17, 28–30]) also failed to establish a strong association between striatal D2/3R availability and BMI, just a modest negative association [17]. Interestingly, two of the articles included used same individuals [28, 30] but showed opposite contributions to the results of this meta-analysis[17]. In Haltia et al. (2007) [30], the subjects performed a blind placebo, and in Haltia et al. (2008) [28], they performed an open placebo [28, 30]. In the blind placebo, subjects were told they would receive either glucose or a placebo, but all received a placebo. On the other hand, in the open placebo, subjects knew they would do a placebo. A blind placebo contributes to a lowered D2R availability in overweight individuals, whereas an open placebo contributes to a higher availability, a response that seems to be related to expectation. Future meta-analyses on this issue using this radiotracer should consider this aspect during the selection of the studies. A preprint study using [^18^F]fallypride and [^11^C]raclopride also reinforced the ability of [^11^C]raclopride to reveal differences resulting from dopamine tone [55]. This study using the same individuals with a mean BMI of 29.5 $\pm$ 7.5 kg/m^2^ found a negative correlation with BMI using PET with [^11^C]raclopride, but none for [^18^F]fallypride [55]. Facing these results, the authors concluded that D2/3R BP_ND_ values measured by PET with [^11^C]raclopride are more sensitive to revealing between-group differences resulting from dopamine tone [55]. One possible explanation can be the lower affinity (26 nmol/L) of [^11^C]raclopride to D2/3R compared to [^18^F]fallypride (33 pmol/L) (see Table 3 for details) [56]. Finally, Pak et al. (2023) reported that in subjects with a BMI of 25 kg/m^2^ or higher, D2/3R availability was negatively associated with BMI (4 studies included [7, 17, 29, 31]) [19]. The results of this meta-analysis, which identify differences within a specific BMI range, support the idea that variations in D2/3R availability in obesity may only occur at certain degrees of obesity.

#### [^18^F]fallypride (n = 4)

A study including a large sample of male and female subjects (n = 130) from underweight to class III obesity concluded that BMI was not associated with D2/3R BP_ND_ in subjects under 30 years old (n = 73) in the midbrain, caudate, putamen, and vSTR [22]. However, it was positively associated with D2/3R BP_ND_ in the same regions among subjects over 30 years old (n = 57) [22]. However, only 18 out of 130 participants were obese, and only 3 of them presented a BMI higher than 40 kg/m^2 ^[22]. Moreover, the mean BMI of this study is in the overweight range (25.5 ± 4.8 kg/m^2^), and all 3 subjects with a BMI higher than 40 kg/m^2^ were over 30 years old [22]. Across all ages (35.60 $\pm$ 18.2 years old), BMI demonstrated a positive association with D2/3R BP_ND_ only in the putamen [22]. Dunn et al*.* (2019) [8], when investigating a female population with a similar combined mean age of 39.615 $\pm$ 9.543 years old but a much higher mean BMI for obesity, 39 $\pm$ 6 kg/m^2^, reported that BMI shows a significantly positive association with D2/3R BP_ND_ not just in putamen but also in caudate. In addition, the authors reported that D2/3R BP_ND_ in obesity was significantly higher in caudate, putamen and vSTR[8]. On the contrary, Guo et al*.* (2022) [49] and Kessler et al*.* (2014) [36] failed to support evidence towards higher striatal D2/3R BP in class I/II obesity. These two studies, including subjects with a younger combined mean age of around 30 (29.28 $\pm$ 3.37 years old), reported that BMI values present a borderline significant negative correlation with D2/3R BP_ND_ in left caudate and right amygdala but none in the putamen, vSTR, substantia nigra (SN), right caudate and left amygdala[36] and D2/3R BP_ND_ did not differ between participants with and without obesity [49]. Finally, when the meta-analysis of Ribeiro et al. (2023) took together two [^18^F]fallypride PET studies [21, 22], also reported no differences in striatal D2R availability between obesity and controls. The article of Guo et al. published in 2014 [21] included in the meta-analysis of Ribeiro et al. (2023) [18] was updated in 2022 [49].

#### [^123^I]IBZM (n = 3)

All [^123^I]IBZM studies found lower D2/3R BP_ND_ in class III obesity compared to the normal-weight group [25–27]. Still der Zwaal et al*.* (2016) and van de Giessen et al*.* (2014) reported no correlation between BMI and D2/3R BP_ND_ in the striatum [26, 27]. All these studies investigate only females with a mean BMI in class III obesity [25–27]. Ribeiro et al. (2023), when investigating the same [^123^I]IBZM articles, evidently came to the same results and conclusion [18]. These studies support the hypothesis that altered D2/3R function may only be evident in obese individuals with a BMI higher than 50 kg/m^2^, as all included individuals are in this BMI range. In addition, the fact that this radiotracer is susceptible to endogenous dopamine and presents a lower affinity to D2/3R compared to the other radiotracers (see Table 3 for details) supports the link between dopaminergic tone and the degree of obesity [33].

#### [^11^C]( +)PHNO (n = 2)

Finally, we will discuss the studies that used [^11^C]( +)PHNO, the only agonist radioligand of the studies retained. Agonist ligands could be more sensitive for revealing between-group differences, especially among subjects with lower BMI, as shown in a study using both antagonist and agonist ligands to examine the association between BMI within the non-obese range [31]. In the vSTR, the authors found a positive correlation between BMI and central dopamine receptor binding values measured by [^11^C]( +)PHNO (agonist) but no relationship using [^11^C]raclopride (antagonist) [31]. We add that [^11^C]( +)PHNO presents a higher susceptibility to endogenous dopamine compared to [^11^C]raclopride (see Table 3). One of the retained [^11^C]( +)PHNO studies, including normal-weight, overweight and obese male and female subjects with a combined mean age of 36.20 $\pm$ 10.41 years old and a mean BMI in class II obesity, found that BMI was positively associated with D2/3R BP_ND_ in the SN/ventral tegmental area (VTA), vSTR and pallidum [32]. The higher tracer binding found in regions densely populated with D3R might be because [^11^C]( +)PHNO has been shown to have higher binding in D3R-predominant extrastriatal regions compared with D2R-predominant dorsal striatal regions (see Table 3) [32, 54, 57]. This result reinforces the previous question of whether obesity-related differences are more prominent in D3R. Cosgrove et al*.* (2015) [37], using the same radiotracer, also found a positive association between class I/II obesity and D2/3R BP_ND_ in the right dorsal caudate. No association was found for the left dorsal caudate [37]. The meta-analysis of Pak et al. (2023) [19] corroborates these findings.

## Conclusion

Results from PET and SPECT studies exploring central dopamine receptors in obesity are not consensual, and most did not link their results to endogenous dopaminergic tone. Here, the distinct characteristics of radiotracers were used to understand these discrepancies and underpin the role of dopaminergic tone in obesity. While the role of D2/3R density in obesity could not be entirely ruled out, dopaminergic tone seems to have a greater impact on the obesity-related differences found in these receptors. Moreover, we support the hypothesis of a quadratic relationship between dopaminergic tone and degree of obesity.

Radiotracers with non-displaceable binding, such as [^11^C]NMB, do not compete with endogenous dopamine for binding in central dopamine receptors. As such, although they can not be used to link dopaminergic tone to the degree of obesity, they are unbiased when investigating the role of brain dopamine receptor density in obesity. The only [^11^C]NMB study retained reported no differences in striatal D2R availability between class II obesity and controls. More clinical studies using radiotracers with these characteristics are needed. Results from animal work investigating these receptors in obesity are not entirely consensual. A mouse model of D2R over-expression showed that the overexpression of these receptors during development persistently increases the propensity for obesity by reducing energy output in mice [58]. Another pre-clinical study reported that D2R down-expression reduced food intake and body weight while displaying an increased basal energy expenditure level compared with wild-type mice [59]. In contrast, Beeler et al. (2016) found no difference between a mouse model of D2R down-expression and wild-type mice regarding weight gain or appetitive motivation in a standard environment [60]. However, in an enriched environment with voluntary exercise opportunities, the model of D2R down-expression exhibited dramatically lower activity and became more obese than wild-type mice, obtaining no protective benefit from exercise opportunities [60]. Other authors also concluded that low striatal expression of D2R contributes to physical inactivity in obesity but did not contribute to a higher vulnerability to diet-induced weight gain than control mice [61]. A review investigating the bidirectional effect of exercise on dopamine signalling reported that exercise increased urine dopamine, striatal dopamine D2/3R availability, dopamine release in the caudate nucleus and in the ventromedial striatum, and blood plasma dopamine. Physical inactivity appears to contribute to impaired dopamine tone, suggesting that dopaminergic tone may play a more significant role in obesity than brain dopamine receptor density.

All studies using radiotracers with displaceable binding, including healthy-weight and individuals with BMI values over 51 kg/m^2^, found lower binding of these receptors in the obese group compared to the normal-weight group [25–27, 50]. These results go with the overall investigation on this topic, proposing lower striatal D2/3R binding for higher degrees of obesity [3, 10, 18]. Moreover, as the radiotracers used in these studies, [^123^I]IBZM and [^11^C]raclopride, are susceptible to endogenous dopamine (see Table 3), these results also support the link between dopaminergic tone and the degree of obesity [33]. In addition, they support the hypothesis that lowered D2/3R availability in obesity might be an exception restricted to BMI higher than 50 kg/m^2 ^[17]. Regarding class I/II obesity, the studies were not as consistent. The [^11^C]( +)PHNO was the only radiotracer where all studies supported the evidence towards higher striatal D2/3R BP in class I/II obesity [3, 10]. Given the higher affinity of [^11^C]PHNO for D3R, we question whether obesity-related differences are more prominent in D3R. We add that [^11^C]( +)PHNO presents the highest susceptibility to endogenous dopamine compared to the other radiotracers (see Table 3), reinforcing the potential role of dopaminergic tone in the obesity-related differences found in these receptors. The negative results of [^11^C]raclopride studies could be attributed, assuming the hypothesis of a quadratic relationship between dopaminergic tone and degree of obesity, to the class III obesity effects cancelling the ones of class II [17, 51]. Another reason highlighted was the potential bias introduced by the menstrual cycle when studying small sample sizes of women of reproductive age [29]. The preprint study discussed in the previous section [55] and the meta-analysis of Karlsson et al. (2015) [17] led us to reinforce the notion that radiotracers with a higher susceptibility to endogenous dopamine or with a lower affinity to D2/3R, such as [^11^C]raclopride, appear more sensitive to reveal between-group differences concerning dopamine tone. Thus, the two [^18^F]fallypride studies retained [36, 49] that did not fit the hypothesis linking dopaminergic tone to the degree of obesity may be explained by radiotracer characteristics. Radiotracers such as [^18^F]fallypride, which are less sensitive to endogenous dopamine or have a higher affinity for D2/3 receptors, seem more suitable for detecting differences in brain dopamine receptors density.

In sum, despite obesity severity, this study highlights the influence of radiotracer characteristics when assessing D2/3R. The hypothesis tested proved to be more suitable for radiotracers that are more susceptible to endogenous dopamine or have a lower affinity to D2/3R, supporting the quadratic relationship between dopaminergic tone and degree of obesity. However, the cut-off value for lowered D2/3R availability in obesity might be 50 kg/m^2^ instead of the proposed 40 kg/m^2 ^[3, 18]. While the role of D2/3R density in obesity could not be entirely ruled out, dopaminergic tone seems to have a greater impact on the obesity-related differences found in these receptors. Finally, neuropsychological factors such as eating or reward behaviours should be tested in addition to BMI, as they may better reflect altered brain dopaminergic function.

## Data Availability

Not applicable. Since this is a systematic review data are available from the original articles. Metadata are included in the current manuscript.

## Abbreviations

BMI: Body mass index
BP: Binding potential
BP_ND_: Nondisplaceable binding potential
D2R: Dopamine type 2 receptors
D3R: Dopamine type 3 receptors
D2/3R: Dopamine type 2 and 3 receptors
PET: Positron emission tomography
ROI: Region of interest
SN: Substantia nigra
SPECT: Single-photon emission computed tomography
VTA: Ventral tegmental area
vSTR: Ventral striatum
WL: Weight loss
[^11^C]NMB: (N-[^11^C]methyl)benperidol
[^123^I]IBZM: [^123^I]iodobenzamide

## Glossary. In the context of this article

• Affinity: refers to the strength or binding capability of the radiotracer for dopamine receptors in the brain
• Availability: refers to the number and accessibility of dopamine receptors in the brain for specific signalling molecules, such as radiotracers
• Displaceable/non-displaceable binding: radiotracers can be categorised into two main types based on their binding characteristics: radiotracers with displaceable binding and radiotracers with non-displaceable binding. Radiotracers with displaceable binding are compounds that bind reversibly to receptors. On the other hand, radiotracers with non-displaceable binding bind irreversibly or with a very high affinity to receptors. Once these radiotracers bind to dopamine receptors, they do not readily dissociate or release, making their binding stable. Therefore, radiotracers with displaceable binding compete with endogenous dopamine for binding in the same receptors, whereas radiotracers with non-displaceable binding are not influenced or affected by endogenous dopamine
• Susceptibility: refers to the likelihood of the radiotracer being influenced or affected by the presence of naturally occurring dopamine in the brain, the endogenous dopamine
